# The economic impact of sight loss and blindness in the UK adult population

**DOI:** 10.1186/s12913-018-2836-0

**Published:** 2018-01-30

**Authors:** Lynne Pezzullo, Jared Streatfeild, Philippa Simkiss, Darren Shickle

**Affiliations:** 1 Deloitte Access Economics Pty Ltd, 8 Brindabella Circuit, Canberra Airport, 2609 ACT Australia; 2 Royal National Institute of Blind People, London, UK; 3 Leeds Institute of Health Sciences, University of Leeds, London, UK

**Keywords:** Sight loss, Blindness, Visual impairment, Cost-of-illness, Health economics, Cost analysis, United Kingdom

## Abstract

**Background:**

To quantify the economic impact of sight loss and blindness in the United Kingdom (UK) population, including direct and indirect costs, and its burden on health.

**Methods:**

Prevalence data on sight loss and blindness by condition, Census demographic data, data on indirect costs, and healthcare cost databases were used. Blindness was defined as best corrected visual acuity (BCVA) of < 6/60, and sight loss as BCVA < 6/12 to 6/60, in the better-seeing eye.

**Results:**

Sight loss and blindness from age-related macular degeneration (AMD), cataract, diabetic retinopathy, glaucoma and under-corrected refractive error are estimated to affect 1.93 (1.58 to 2.31) million people in the UK. Direct health care system costs were £3.0 billion, with inpatient and day care costs comprising £735 million (24.6%) and outpatient costs comprising £771 million (25.8%). Indirect costs amounted to £5.65 (5.12 to 6.22) billion. The value of the loss of healthy life associated with sight loss and blindness was estimated to be £19.5 (15.9 to 23.3) billion or £7.2 (5.9 to 8.6) billion, depending on the set of disability weights used. For comparison with other published results using 2004 disability weights and the 2008 estimates, the total economic cost of sight loss and blindness was estimated to be £28.1 (24.0 to 32.5) billion in 2013. Using 2010 disability weights, the estimated economic cost of sight loss and blindness was estimated to be £15.8 (13.5 to 18.3) billion in 2013.

**Conclusions:**

The large prevalence of sight loss and blindness in the UK population imposes significant costs on public funds, private expenditure, and health. Prevalence estimates relied on dated epidemiological studies and may not capture recent advances in treatment, highlighting the need for population-based studies that track the prevalence of sight-impairing eye conditions and treatment effects over time.

**Electronic supplementary material:**

The online version of this article (10.1186/s12913-018-2836-0) contains supplementary material, which is available to authorized users.

## Background

Sight loss and blindness cause a considerable amount of health burden globally. In the UK, the burden due to sight loss and blindness has increased from around 143,600 to more than 154,600 disability adjusted life years, while the total disability adjusted life years due to all causes in the UK have been decreasing [1]. Demographic ageing is leading to a substantial increase in the prevalence of age-related sight-impairing conditions, and associated increases in their costs [2]. However, a significant proportion of sight loss and blindness is preventable, suggesting that more investment in prevention and early intervention could improve socioeconomic outcomes in the UK [3].

Estimating the cost of sight loss and blindness is essential if their socioeconomic impact is to be fully understood and if the cost effectiveness of prevention and treatment is to be calculated. Calculation of the economic cost in the UK is necessary as an input to assist decision makers to evaluate policy and to prioritise health expenditure, including research expenditure. Moreover, it is necessary to understand how certain costs are changing over time, and whether any progress has been made against key objectives, including eliminating preventable sight loss and blindness through effective interventions such as screening and early intervention with treatments.

To date, there has been little work estimating the economic impact of sight loss and blindness specifically in the UK context [2], although a study for the Royal National Institute of Blind People exists that estimates the prevalence and cost of sight loss and blindness in 2008 [4]. Previous peer-reviewed studies have aimed to identify the unit costs of sight loss and blindness for use in cost-effectiveness analysis [5], with limited focus placed on estimating the overall impact.

Internationally, a substantial body of literature exists that estimates various costs associated with sight loss and blindness, including direct medical costs, indirect costs and intangible costs. A recent paper summarised 22 cost of illness and intervention studies, including one study in the UK, finding that the mean annual expenses per person for sight loss and blindness range from US$12175 to US$24180 depending on severity [6]. However, very few cost of illness studies related to sight loss or blindness have been published in peer-reviewed literature, and none have been published within the UK context. In Canada, the socioeconomic impact of sight loss and blindness was estimated to be £26,587 per person with sight loss or blindness [7]. In Japan, the cost was estimated to be £28,672 per person with sight loss or blindness [8]. Both values were converted using purchasing power parity in 2007.

This research has calculated the prevalence and cost of sight loss and blindness specifically for the UK context in 2013, to provide more current estimates of the various cost components and to compare the changes over the five years since the original Access Economics study was conducted for the UK. This research enables unique comparisons of the socioeconomic impact of sight loss and blindness across countries by using a methodology consistent with work previously conducted in Canada, Australia, Japan and the US. Moreover, this research provides useful analysis of the changing cost of sight loss and blindness within the UK. As such, this research will help inform decision-making with regards to policy and commissioning of effective interventions for prevention, detection, treatment and care of sight loss and blindness and to provide direction for future research.

## Methods

The costing methodology used in this study is based on a prevalence approach to cost measurement [9]. Prevalence approaches measure the number of people with a given condition in a base period and the costs associated with treating them, as well as other financial and non-financial costs in that year due to the condition. This approach is combined with both top-down and bottom-up approaches to estimate expenditure for each condition. [9]. To identify materials of relevance to this study, a literature review was conducted for each cost component, noting this review was targeted rather than systematic since the former was considered more fit-for-purpose given this was a cost of illness study where many of the inputs are in data sets and grey literature (e.g. government documents) not in peer-reviewed literature.

### Prevalence

To determine prevalence, sight loss and blindness for this research are defined as:

- blindness (severe sight loss) is defined as best corrected visual acuity (BCVA) of < 6/60 in the better-seeing eye; and
- sight loss is defined as BCVA of < 6/12 to 6/60 in the better-seeing eye.

A number of data sources, both local and international, were utilised to estimate the prevalence of sight loss and blindness by age, gender, ethnicity, severity and major cause (Additional file 1). Prevalence rates were adjusted for comorbidity using relativities of each condition to total sight loss and blindness. Total sight loss and blindness data by cause were derived by five year age groups. Within age groups, the relativities between the visual acuity groupings were used to separate total prevalence. For example, for those aged 75 and above the Medical Research Council (MRC) trial of assessment and management of older people in the community was used to establish overall prevalence [10], including the relativities between BCVA < 6/18, 6/18–6/30, and < 6/60 – the visual acuity groupings reported in the study. [11] The relativities of each condition were then applied to the total prevalence to adjust for comorbidities [11–14]. The prevalence rates for the other age groups were derived in a similar way. Ethnicity splits [15–22], calculated based on relative risks for specific eye disease type, were applied to the prevalence rates by age. Where these were not available, prevalence rates were assumed to be the same as for the general population (i.e. no difference by ethnicity group). Detailed tables and methodology outlining the prevalence rate estimates by age, gender and condition are available in the supplementary file (Additional file 1).

These derived prevalence rates were applied to population estimates by age, gender, ethnicity and region from the 2011 UK Census and sub-national population projections for government office regions developed by the Office for National Statistics (ONS). [23, 24] Greater London Authority projections of the London population by ethnicity, gender and five-year age group between 2013 and 2041 were utilised to project ethnicity groups to 2051 across the entire UK population as they were the only publicly available projections that provided age and gender breakdowns for ethnic groups [25]. Average annual growth rates between 2013 and 2041, calculated for each ethnicity group and by five-year age groups, were assumed to apply for the period between 2041 and 2051. The total projected population, by each age and gender group were adjusted to coincide with ONS population projections [26], with each ethnicity group maintaining the same relative share of the total population.

### Health care system expenditure- direct costs

Direct health system costs were determined using a combination of a top down and bottom up approach. [9] The top down approach is based on Reference Cost data collected by the Department of Health in England [27]. Health care services within the Reference Cost data are broken down into Healthcare Resource Groups (HRGV.4+) and contain 58 HRG codes that specifically related to eye disease. To determine hospital inpatient expenditure, each HRG code was mapped to each condition. Alternative sources such as Scotland’s Health Service Costs [28], Wales’ Health Statistics Wales [29], and Northern Ireland’s Reference Costs [30] were used for other devolved nations. Non-admitted expenditure was also estimated using these data. Non-admitted expenditure consists of outpatient costs and other community services, including paramedic services, consultant led outpatient attendances and non-consultant led outpatient attendances.

Other health system expenditure was constructed using a ‘bottom up’ approach [9]. This includes costs associated with sight loss and blindness from prescribing expenditure, general ophthalmic services, injurious falls, research and development (R&D), residential and community care, and capital and administration.

Expenditure data associated with prescriptions such as ranibizumab (Lucentis), aflibercept (Eylea), anti-infective eye preparations, corticosteroids, mydriatics, cycloplegics, local anaesthetics and other ophthalmic preparations was sourced from each devolved nation’s statistical reports as appropriate using national formulary classifications to ensure the prescription is related to sight loss or blindness [31–34]. For example, in England this is the British National Formulary [31].

Similarly, data on general ophthalmic services [35–38], residential and community care attributable to sight loss or blindness [28, 39–41], and capital and administration attributable to sight loss or blindness [29, 42–44] were also sourced from each devolved nation’s statistical reports as appropriate. Residential and community care data sources indicate the number of recipients with sight loss or blindness. To estimate the total cost of residential and community care services, average unit costs for residential and community care – derived by dividing total expenditure by total services – were applied to each residential and community care service. To attribute capital and administration costs to sight loss and blindness, it was assumed that total capital and administration expenses are incurred at the same rate as the overall health expenditure in England, of which 2.0% is for sight loss and blindness [45].

The government sources outlined do not provide sufficient information to estimate the private health care expenditure associate with sight loss. As the public versus private health care expenditure split for health services has been relatively stable over the past two decades with public spending only rising 3% of the total expenditure by 2015 [46], private health care expenditure was derived using data from Williams et al. (2000) [47]. Expenditure associated with injurious falls due to sight loss and blindness was derived using hospital episode statistics [48] and a model developed by Scuffham et al. (2002) [49].

R&D expenditure attributable to sight loss and blindness was estimated by applying the share of eye and ear health R&D [50] to total health related R&D investment from private industry, non-profit organisations and public funds through the government [51]. To separate eye and ear health R&D, expenditure for eye related R&D was assumed to maintain the same proportion as the estimated disease burden attributable to ear and eye health from the World Health Organization (WHO) Global Burden of Disease project for high income countries [52]. This measure was also validated against the most recent Global Burden of Disease project for the UK, which showed that the measure was not sensitive to changes over time with the measure being 29.1% using the more recent approach rather than 29.8% using the earlier approach for high income countries [1, 52].

### Indirect costs:

There are two types of indirect costs of sight loss and blindness: the financial costs associated with lower productivity from premature mortality, lower workforce participation and absenteeism, and the cost of informal carers, aids and modifications and deadweight losses; and the non-financial costs from loss of healthy life, that are analysed in terms of disability adjusted life years (DALYs).

In evaluating indirect costs, it is important to make the economic distinction between real costs and transfer payments. Rather than payments made for the use of any good or service, transfer payments are a transfer of claims over real resources. Transfer costs are important to estimate in order to attribute who bears the costs of sight loss and blindness, and to calculate the deadweight loss to society.

A human capital approach is adopted to estimate productivity losses [9, 53]. Employment rates are lower for people with sight loss relative to the average person in the UK (after age standardisation) [54]. It is assumed that, in the absence of sight loss, people with sight loss would participate in the labour force and obtain employment at the same rate as other people in the UK and earn the same average weekly earnings.

To estimate premature mortality rates from sight loss and blindness in 2013, the country specific mortality rate [55–57] was multiplied by an odds ratio of 2.34, which was derived from the Melbourne Visual Impairment Project (MVIP) [58]. Deaths due to sight loss and blindness by age and gender were calculated from the demographic data and mortality rates utilising the attributable fraction approach.

The productivity loss from those who die prematurely was estimated based on the assumption that if they had lived, the person would have earned an average annual income up until their retirement. Average gross annual incomes were calculated as £29,297 for males and £23,946 for females in England, £26,702 for males and £21,939 for females in Wales, £28,304 for males and £23,608 for females in Scotland, and £24,825 for males and £22,958 for females in Northern Ireland [59]. No on-costs have been included in calculating productivity losses.

Retirement age was represented by the State Pension age. The eligibility age for the State Pension is increasing in the UK over the next decade. For the purposes of estimating the productivity loss associated with premature mortality, this was set at the 2013 eligibility age, which was 65 for males and 62 for females. Average life expectancy was assumed to be 79 for males and 83 years for females. For the age brackets 75–84 and 85+ for both males and females, life expectancy was assumed to be 85 years and 90 years respectively [60].

The number of people who were in employment at the time of their death was calculated by multiplying the number of deaths due to sight loss or blindness by the employment rate of those with ‘difficulty seeing’, which on average was 55.5% [54]. The present value of lost earnings (gross) was calculated using a discount rate of 3.5% over the number of years until retirement with sensitivity analysis at 1.5%, as recommended by the National Institute for Health and Care Excellence [61].

In addition to workforce separation, people with sight loss or blindness may be absent from work more often due to their condition. Access Economics (2006) [62] estimated that people with sight loss or blindness in the United States were likely to have an additional 4.1 days off work per year on average [7, 8]. Productivity costs from increased absenteeism were calculated as the average number of days absent per year due to sight loss or blindness adjusted for employment [7, 8].

Total informal care costs were calculated based on a top-down approach using the 2011 Census data to determine the number of informal care hours provided to people with sight loss, adjusted for population growth to 2013. An opportunity cost methodology [62–64] was used to value these hours, which measures the value in alternative use of time spent caring. This is valued by productivity losses (or value of leisure time) associated with caring.

The total number of hours of informal care were calculated with the same methodology used by the University of Leeds in valuing informal carers for Carers UK [65]. The cost of informal care related to sight loss and blindness was estimated as the total number of informal care hours multiplied by the average per hour wage rate for males and females, derived from the average annual incomes [59].

The total annual cost of devices and modifications was derived from data from a study conducted by Lafuma et al. (2006) [66] in 2004. Prices were converted into Sterling by applying the exchange rate used within their study (£1 = €1.5) and adjusted to 2013 prices using an average UK inflation rate over that period of 2.71% [67].

Real costs use up resources, or reduce the economy’s overall capacity to produce goods and services. In contrast, transfer payments involve payments from one economic agent to another and include taxation revenue or social welfare payments, which impose deadweight losses on society. Deadweight losses associated with sight loss and blindness include the cost of raising additional revenue to fund public health care system costs, residential and community care, aids and equipment, and direct payments to those with sight loss and blindness and their carers.

While the costs associated with deadweight loss depend on the method used to raise additional taxes, the social cost is not zero and has therefore been included as a cost of sight loss and blindness. This study assumes that additional taxes are raised through income tax rate changes. The average marginal cost of raising additional tax revenue was calculated as 1.12 [68], so for every additional £1 raised by the UK government to fund costs associated with sight loss and blindness, there is an estimated £0.12 of deadweight loss. Direct payments from the government to those with sight loss were sourced from the Department for Work and Pensions [69] and the Department of Social Development [70] for Northern Ireland.

### Burden of disease

The overall impact on wellbeing from disability and premature death can be measured as “burden of disease”, measured in DALYs. DALYs have two components – the years of healthy life lost due to disability (YLD) and the years of life lost due to premature death (YLL).

The method to quantify the reduction in the stock of health capital is the global burden of disease methodology developed by the WHO [71].

In any year, the disability weight of a disease (for example, 0.43 for blindness) reflects a relative health state. This example represents losing 43% of a year of healthy life because of blindness. The loss of wellbeing for sight loss was estimated using disability weights as per the global burden of disease study from 2004 and 2010, noting the existing debates surrounding the methodologies used to generate each set of disability weights [72–76]. Both sets of disability weights are validated measures and undergo considerable peer review at the time of publication. Presenting the loss of wellbeing using both sets of disability weights has added benefits of enabling comparison with not only the earlier Access Economics study, but also new studies going forward that are based on the 2010 disability weights. Each set of disability weights was applied to prevalence data to calculate DALYs.

The method to value a reduction in the stock of health capital has been based on Mason et al. (2008) [77] using estimates of the value of a statistical life (VSL) derived from the UK Department of Transport. The UK Department of Transport estimated the VSL to be £1.43 million in 2005 prices. Mason et al. estimated, the value of a year of perfect health to be £70,896 (in 2005 prices), by applying a discount rate of 1.5%. This is the rate of pure time preference and differs from the standard 3.5% discount rate as the VSL has been shown to grow at a similar rate as the marginal utility of consumption (approximately 2%), and should therefore be excluded [77]. This estimate for the value of a DALY was adjusted to 2013 prices using UK consumer price index to give £88,825.

## Results

### Prevalence

In 2013, there were an estimated 1.93 (1.58 to 2.31) million people with sight loss and blindness in the UK as a whole, or 3.0% (2.5% to 3.6%) of the population. This includes 255,000 (208,100 to 304,800), or 13.2% who are blind. The ethnic groups with highest prevalence were white people (3.3%, range 2.7% to 3.9%), followed by Asians (1.5%, range 1.2% to 1.8%). The prevalence of sight loss and blindness in the UK was estimated to have increased by 135,115 (7.5%) since 2008.

The prevalence of sight loss and blindness is projected to increase with demographic ageing, and in a policy neutral environment, from 3.0% (2.5% to 3.6%) today to 5.4% (4.4% to 6.5%) or approximately 4 million people by 2050. In terms of ethnic shares relative to the total population with sight loss and blindness, the share of white people with sight loss and blindness is projected to fall (from 94.9% to 88.5%), while the share of black people is projected to increase from 1.0% to 1.8%, Asians from 3.0% to 6.4%, and other ethnicities from 1.1% to 3.3%.

From 2013 to 2050, the share of sight loss and blindness from AMD is projected to change from 23.1% to 29.7%, more than doubling from 445,809 (363,900 to 532,800) people to 1.23 (1.01 to 1.47) million people. The share contributed from cataract is projected to change from 18.7% to 21.4%, diabetic retinopathy from 4.7% to 3.1%, glaucoma from 7.2% to 7.0%, under-corrected refractive error from 38.9% to 31.3% while other eye diseases remains constant in its share of total prevalence (rising in absolute terms only).

### Health system costs- direct costs

Direct health care system costs are estimated to amount to £2.99 billion in UK in 2013. Around 50% of total direct health care system costs are attributable to hospital recurrent expenditure and non-admitted expenditure, totalling around £1.5 billion. Further significant cost items include general ophthalmic services (£614.6 million or 21%), prescribing expenditure (£380.9 million or 13%), and residential and community care services (£276.8 million or 9%). Other costs include costs due to injurious falls, an attributable portion of capital and administration costs and research and development relating to sight loss and blindness (Table 1).

**Table 1:** Summary of health care system expenditure, by country 2013, £ million and % of total

	England	Wales	Scotland	Northern Ireland	UK
Hospital recurrent expenditure	588.9, 23.9%	44.7, 29.6%	85.9, 29.4%	15.3, 19.1%	734.9, 24.6%
Non-admitted expenditure	673.8, 27.3%	33.7, 22.3%	47.3, 16.2%	16.3, 20.3%	771.1, 25.8%
* Prescribing expenditure*	137.9, 5.6%	9.0, 6.0%	11.8, 4.0%	3.9, 4.9%	162.6, 5.4%
Ranibizumab (*Lucentis*)^a^	184.0, 7.5%	11.6, 7.7%	17.5, 6.0%	5.2, 6.5%	218.3, 7.3%
General ophthalmic services (GOS)	481.1, 19.5%	32.3, 21.4%	80.9, 27.7%	20.2, 25.2%	614.6, 20.6%
Expenditure associated with injurious falls	19.9, 0.8%	1.0, 0.7%	2.0, 0.7%	0.6, 0.7%	23.4, 0.8%
	(16.2, 0.7% - 23.7, 1.0%)	(0.8, 0.5% - 1.2, 0.8%)	(1.6, 0.5% - 2.4, 0.8%)	(0.5, 0.6% - 0.7, 0.9%)	(19.1, 0.6% - 28.0, 0.9%)
Research and development	14.1, 0.6%	0.5, 0.3%	2.1, 0.7%	0.3, 0.4%	17.0, 0.6%
Residential care and community care services	220.8, 9.0%	13.2, 8.7%	31.7, 10.9%	11.0, 13.7%	276.8, 9.3%
Capital and administration	145.7, 5.9%	5.1, 3.4%	12.8, 4.4%	7.1, 8.9%	170.7, 5.7%
Total	2466.1	151.1	292.1	80.1	2989.3
	(2462.4–2469.9)	(150.9–151.3)	(291.7–292.4)	(80.0–80.2)	(2985.0–2993.9)

^a^Ranibizumab (Lucentis) sales data for the UK has been split based on the estimates of prevalence for AMD

AMD accounted for 34% of total health system costs that could be attributed to each of the five conditions (this total excludes residential care and community care services, expenditure associated with injurious falls and capital and administration expenditure). This represents the rapid growth in costs associated with the new anti-vascular endothelial growth factor (VEGF) therapies such as ranibizumab (Lucentis) and Aflibercept (Eylea). Under-corrected refractive error, cataract, diabetic retinopathy, glaucoma and other eye diseases accounted for 21%, 20%, 10%, 7% and 8% of total health system costs, respectively.

### Indirect costs

The total of indirect costs attributable to sight loss and blindness in the UK were estimated to be £5654 (5117 to 6224) million in 2013. Indirect costs for each country are shown in Table 2.

**Table 2:** Summary of indirect costs 2013 (£ million)

	England	Wales	Scotland	Northern Ireland	UK
Lower employment	2078.5	88.0	210.9	50.0	2427.4
	(1696.5–2484.2)	(71.8–105.2)	(172.2–252.1)	(40.8–59.8)	(1981.2–2901.3)
Absenteeism	65.6	3.7	6.5	1.9	77.6
	(53.5–78.4)	(3.0–4.4)	(5.3–7.8)	(1.6–2.3)	(63.4–92.8)
Premature mortality	1.77	0.09	0.23	0.05	2.14
	(1.44–2.11)	(0.08–0.11)	(0.19–0.27)	(0.04–0.07)	(1.75–2.56)
Informal care costs	1951.9	134.8	194.8	76.7	2358.2
	(1951.9–1951.9)	(134.8–134.8)	(194.8–194.8)	(76.7–76.7)	(2358.2–2358.2)
Devices and modifications	343.8	21.5	34.1	10.2	409.6
	(280.6–411)	(17.6–25.7)	(27.8–40.8)	(8.3–12.2)	(334.3–489.6)
Deadweight loss	311.8	19.8	37.0	10.3	379.0
	(311.4–312.2)	(19.8–19.8)	(37–37.1)	(10.3–10.3)	(378.5–379.4)
Total	4753.3	267.8	483.6	149.2	5653.9
	(4295.4–5239.8)	(247.0–290.0)	(437.3–532.8)	(137.7–161.3)	(5117.4–6223.9)

Lower employment participation for those with sight loss and blindness resulted in 90,108 (73,500 to 107,700) fewer people in the UK workforce in 2013 and an estimated loss of £2.43 (1.98 to 2.90) billion in income (44% of indirect costs).

The second largest indirect cost component is attributable to informal care, estimated to be around £2.36 billion (or 43%) in 2013. Other indirect costs associated with sight loss and blindness in 2013 include expenditure on devices and modifications (£409.6 million or 7%, range 334.3 to 489.6 million), deadweight loss (£379.0 million or 7%, range 378.5 to 379.4 million), absenteeism and premature mortality (together, 1% of indirect costs). In the working age population, an estimated 25 (20 to 30) deaths were attributable to sight loss and blindness in 2013, of which 14 (11 to 17) would have been employed.

### Burden of disease

**Fig. 1:**
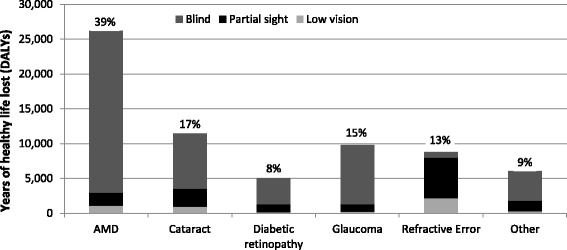
Burden of disease across conditions in the UK, by severity of sight loss, 2013. Note: % refers to the total burden of disease caused by the condition. Burden of disease has been determined using 2004 disability weights. Abbreviations: AMD, age-related macular degeneration; DALYs, disability-adjusted life years

Using disability weights from the 2004 global burden of disease project for comparison with the 2008 estimates and older international studies, the YLDs lost due to sight loss and blindness were estimated as 205,372 (167,600 to 245,500) DALYs in the UK in 2013. Based on the number and age-gender profile of deaths, the YLLs from sight loss and blindness were estimated to be 13,734 (11,200 to 16,400), bringing the total to 219,106 (178,800 to 261,900) DALYs. The proportion of DALYs attributable to each condition are shown in Fig. 1.

Total DALYs were multiplied by £88,825 (the value of a DALY) to provide an estimate of £19.5 (15.9 to 23.3) billion for the total cost associated with the loss of wellbeing. The cost was estimated to be £16.3 (13.3 to 19.5) billion in England, £1.0 (0.8 to 1.2) billion in Wales, £1.6 (1.3 to 1.9) billion in Scotland and £0.5 (0.4 to 0.6) billion in Northern Ireland.

Applying the alternative 2010 global burden of disease disability weights – for comparison with newly published cost of illness studies that adopt these weights – would result in a substantially lower estimate of 81,033 (66,100 to 96,900) DALYs from sight loss and blindness, with the value of the loss of wellbeing estimated to be £7.2 (5.9 to 8.6) billion in the UK in 2013.

## Discussion

The results of the study indicate that sight loss and blindness in the adult population places a large economic cost on the UK, estimated to total £28.1 (24.0 to 32.5) billion in 2013 using 2004 disability weights, or £15.8 (13.5 to 18.3) billion using 2010 disability weights. The former estimate provides useful comparison with 2008 estimates and work undertaken in Canada and Japan, which use similar disability weights. The per capita cost of sight loss and blindness was estimated to be £14,549, which is lower than both Canada and Japan which were £26,587 and £28,672 respectively when converted using purchasing power parity in 2007 [7, 8].

Comparing 2008 and 2013, the cost of sight loss and blindness in the UK is estimated to have increased by 27.8%. The increase is driven by higher prevalence as well as an estimated 39% increase in health system expenditures, including a more than doubling of prescribing expenditures due to the inclusion of the cost of ranibizumab (Lucentis) and over a 50% increase in non-admitted expenditures. However, residential and community care costs associated with sight loss and blindness have fallen over the period as the rate of accessing these services for people with sight loss or blindness has fallen relative to 2008. People with sight loss and blindness have been disproportionately affected, with the rate of accessing services declining by more than for other disability groups [78]. This may be both a result of a decline in the number of people with sight loss or blindness that have a Certificate of Vision Impairment and a result of cuts to social care budgets in the UK from 2010 onwards. Recently, more funding has been committed to increasing social care services and other forms of assistance. For example, the Care Act in England which came into effect in April 2015 legislates that Local Authorities must provide minor aids and adaptations up to the value of £1000 for the purpose of assisting with nursing at home or aiding daily living, which would have implications for expenditure in the future.

The estimated 49% rise in productivity losses was due to a fall in the employment rate for people with sight loss and blindness, thereby widening the employment gap compared to the general population. This also directly contributed to the fall in the cost associated with absenteeism which is dependent on the number of employed people with sight loss and blindness.

With population ageing, the prevalence of AMD is estimated to have risen nearly 49% over the period 2008 to 2013. The ageing population and increasing prevalence of diabetes in UK is associated with estimated prevalence increases of over 40% for cataract, glaucoma and diabetic retinopathy. However, the prevalence of sight loss and blindness due to under-corrected refractive error has decreased over the same period, possibly due to more people accessing corrective services as the value of vouchers for accessing corrective glasses and the number of eye tests has increased.

Importantly, the burden of disease from sight loss and blindness is estimated to have increased by more than 25% compared with 2008, indicating that the substantial burden of disease from sight loss and blindness is still growing in the UK.

While unavoidable, age-related conditions contribute greatly towards the growing economic costs and burden of sight loss and blindness in the UK; although, a substantial proportion of this is still preventable. For example, it has been estimated that the majority of visual impairment worldwide, including blindness, may be preventable using cost-effective treatment methods that are already available. Further, WHO Member States (including the UK) have committed to reducing the prevalence of avoidable visual impairment by 25% by 2019 compared to the baseline established by the WHO in 2010 [3].

To align with data sources, the definitions of sight loss and blindness in this research differ slightly from the guidelines for certifiable severely sight impaired (blindness) and sight impaired (partial sight), which are [79]:

- severely sight impaired is defined as BCVA of < 3/60, or those > 3/60 and < 6/60, and < 6/60 with the presence of contracted field of vision; and
- sight impaired is defined as BCVA of > 3/60 and < 6/60 with full field, or > 6/60 and < 6/24 with moderate contraction of the field, or < 6/18 or even better with marked contraction of visual field.

This means that the prevalence numbers reported here will not align with certifiable sight loss and blindness. While the scope of the exercise was not to report on certifiable sight loss and blindness, it is important to recognise that the economic costs and burden of sight loss and blindness reported in this article are larger than for certifiable sight loss and blindness alone. This distinction is important for policy implications.

There are some limitations to this study. The methods for this study outlined the use of grey literature and a non-systematic search strategy, noting that this was fit-for-purpose. The estimates presented in this article should be interpreted with this in mind. Judgement and experience undertaking cost of illness studies – particularly for sight loss and blindness in the UK [4] and internationally [2, 7, 8, 80] – has been used to ensure that the estimates are as accurate and complete as possible without undermining the quality of the estimates presented.

With reference to health expenditure costs, using a top-down approach to estimate expenditure has advantages of readily available data, simplicity and low cost to conduct analysis. However, top-down approaches rely on accuracy of data recording. For example, when determining direct health system expenditure in hospitals, the top-down approach utilises healthcare resource groups where expenditure is associated with eye conditions as the primary cause. In contrast, a bottom-up approach that uses anonymised patient records linked to hospital episode statistics can track expenditure for individuals identified as having sight loss or blindness regardless of the primary reason for admission, such as falls. However, linked data is often not readily available for research purposes, and this type of approach often requires costly data analysis. Further, a bottom-up approach can still be subject to data recording issues, including in clinical coding data. When considering primary diagnosis data, clinical coding errors occur in approximately 11% of cases, and is higher for secondary diagnosis data [81]. This can lead to poor tracking of expenditure attributed to eye health conditions, and limits the application of a bottom up approach. Noting the limitations of a top down approach, the costs presented in this analysis are estimates, although the methods which they are based on are peer-reviewed [80], and have been utilised in other countries previously [2, 7, 8, 80].

In addition, recent healthcare or policy changes may not be captured appropriately. For example, it was assumed that there have been little to no changes in the private expenditure for sight loss or blindness procedures since 1998 [47]. The estimated private expenditure for procedures was approximately £150 million. If there has been a trend for these procedures to be publically funded as with overall health expenditure [46], there would likely be small downward correction to the total cost of sight loss or blindness in the UK.

To estimate the R&D expenditure attributable to sight loss and blindness, it was assumed that the proportion of health burden due to sight loss or blindness relative to total eye and ear health burden applied to R&D expenditure. In reality, this assumption may be overly simplistic for R&D expenditure, but it does provide a useful starting point. The R&D expenditure was estimated to be £17.0 million, which may or may not fully capture the actual research resources devoted to sight loss and blindness in the UK.

For community and social support services provided to people with sight loss or blindness, unit costs were assumed to be the same as for all service recipients. Also, capital and administration expenditure was assumed to be attributed to sight loss and blindness in a similar proportion to overall health expenditure. These are necessary simplifying assumptions due to a lack of available data. For example, it is still important to include the cost of having surgery equipment and infrastructure to provide cataract surgery. Likewise, a person with sight loss or blindness would likely receive an average level of support from community and social support services.

Another potential limitation is using a human capital approach to estimate productivity losses, which can lead to higher estimates of the cost of unemployment due to sight loss and blindness than alternative approaches such as the friction cost approach [9, 53]. The human capital approach differs to the friction cost approach in that the productivity loss of the worker’s contribution relative to the absence of the condition is included for the duration of the condition, whereas the friction cost approach only includes productivity losses until the worker is replaced [9, 53]. The human capital approach is appropriate for industrialised countries in which there is near full employment as the removal of labour constrains economic growth in the long run production possibilities frontier, ceteris paribus [53, 80]. If the friction cost approach was used, the estimated productivity loss of £2.43 billion would be expected to be considerably smaller.

To estimate the costs of informal care provision, average wages were used. This may be seen to overstate the cost of informal care associated with sight loss and blindness. However, it is important that informal care is valued at average wage rates as this captures the value of lost opportunity of undertaking leisure time, which is proxied using the average age and gender specific wage rate [82, 83]. Moreover, if informal care was not provided, it is likely that formal care would be used as a substitute where available, which would have a higher replacement cost than the estimates presented in this study.

Regarding absenteeism estimates, the estimated days off work and methods are consistent with the methods used by Roberts et al. (2010) [8] and Cruess et al. (2011) [7], which provides useful comparison of the work impacts of sight loss and blindness between Canada, Japan, the United States and the UK. However, while the methods are consistent, the estimates in all studies are now based on older employment information from the United States, and no estimates specific to the UK are available. Furthermore, no recent studies have been identified that attempt to classify absenteeism impacts [6]. Further research surrounding the effect of sight loss and blindness on work should be undertaken.

To estimate ethnicity groups by age and gender to 2050, this research utilised Greater London Authority projections [25]. A number of other methodologies and sources have attempted to provide ethnicity projections over the long term. Prominent projections for the UK suggest that the ethnic minority share of the population would increase from approximately 13% in 2001 to between 20 and 25% [84] to 44% [85] by 2051 and 2056, respectively. This contrasts with minority groups representing approximately 18% of the total UK population by 2050 estimated in this research. A decision was made to use Greater London Authority projections as data was available by year and did not require linear interpolation between 2001 and 2051. It is noted that there is substantial uncertainty surrounding population and ethnicity group projections. Although ethnic composition does not affect prevalence substantially, sensitivity analysis was nonetheless conducted on prevalence estimates to account for this uncertainty. Confidence intervals from prevalence studies were weighted by sample size to estimate a robust prevalence range. The ranges presented in this research reflect a decrease or increase in prevalence by 18.4% and 19.5%, respectively.

Finally, quality adjusted life years (QALYs) are typically used in the UK for cost effectiveness analysis [5, 86]. This means that DALYs are unfamiliar to the UK context. DALYs are recommended in the Vancouver group guidelines to measure the burden of disease due to sight loss and blindness, measuring the disability imposed on an individual or the loss of healthy life years [80]. DALYs have been used in a number of studies to estimate the cost burden of sight loss and blindness internationally, and provide a useful basis for comparison across countries. [2, 82, 87] QALYs often rely on preference-based health related quality of life measures elicited from general population samples or from groups of people with the specific condition (e.g. sight loss or blindness), which can make it difficult to make comparisons internationally or with other conditions [88]. DALYs also use a standard life expectancy tables across countries, which make international comparisons easier [88]. For these reasons, DALYs are appropriate and useful in cost of illness studies.

Going forward, more precise and reliable epidemiological information is required to make better estimates of the total national prevalence and costs associated with sight loss and blindness. As the majority of prevalence studies are almost a decade old, the prevalence estimates may not capture those in non-community settings, people with learning disabilities and dementia, or recent advances in treatment. In particular, the effects of recent growth in the use of anti-VEGF therapies to treat AMD and diabetic retinopathy are unlikely to be taken into account in prevalence estimates, although they were in the cost estimates. Anti-VEGF medicines have been shown to both slow and reverse some instances of sight loss and blindness [89].

Changes in eligibility for sight tests funded by the National Health Service (NHS) may have altered detection rates, which may then have resulted in earlier treatment and a reduction in the prevalence of sight loss and blindness. Specifically, in 2006, NHS Scotland expanded the eligibility criteria for NHS funded eye tests so that the entire Scottish population receives free eye tests. Subsequent adjustments to eligibility in relation to the frequency of free eyes tests were also made. Increased eligibility for comprehensive eye examinations may have benefits from the societal perspective in Scotland if policy encourages a shift in emphasis from the sale of optical appliances to eye health, and if this leads to higher risk individuals receiving eye examinations [90].

These limitations of prevalence data highlight the need for high quality, population-based epidemiological studies to track the prevalence of all eye conditions in the UK and the impacts of treatment over time. Building on the evidence base longitudinally in this manner could also capture changes that are occurring within the population (e.g. in risk factors such as smoking rates), enabling more accurate and timely estimates of the economic costs associated with sight loss and blindness. UK-wide population projections that include ethnicity splits would also be highly beneficial for future studies estimating the economic impact of sight loss and blindness in the UK.

Despite the limitations with recent prevalence data, this study provides an important, and likely accurate picture of the economic costs associated with sight loss and blindness in the UK. To provide some reassurance to the reader that this limitation in prevalence data does not undermine the overall estimates presented in this study, we examined the number of people who are registered as partially sighted or blind (i.e. certifiable sight loss or blindness) with England councils. In 2008, 309,265 people were registered as either partially sighted or blind in England [91]. In 2014, 291,100 people were registered as either partially sighted or blind, a decrease of 6% [91]. The number of people registering with councils may have declined due to changes in care needs, various policy considerations or an overall decrease in prevalence. However, the magnitude of this change indicates that the expected prevalence in 2013 is within the sensitivity analysis conducted, noting that this is on the lower end of the sensitivity analysis at worst.

Overall, the large prevalence of sight loss and blindness means sight loss in the UK adult population imposes a substantial cost on public funds, private expenditure, and health. This study did not estimate the expected large, and additional costs, associated with sight loss and blindness for children (less than 18 years of age) so the total cost is expected to be underestimated.

Data on the prevalence of childhood sight loss and blindness in the UK is limited and variable. More research needs to be undertaken into measuring childhood sight loss and blindness and the associated economic costs within the UK.

## Conclusion

Estimating the cost of sight loss and blindness is essential if their socioeconomic impact is to be fully understood. The large prevalence of sight loss and blindness in the UK population imposes significant costs on public funds, private expenditure, and health – estimated to total £28.1 (24.0 to 32.5) billion in 2013 using 2004 disability weights, or £15.8 (13.5 to 18.3) billion using 2010 disability weights. Prevalence estimates relied on dated epidemiological studies and may not capture recent advances in treatment, or the substantial increase in the prevalence of age-related sight-impairing conditions due to demographic ageing, and associated increases in their costs. Importantly, this study takes account of new data to update the understanding of the economic impact of sight loss and blindness in the UK, enabling international comparison and an estimate of the change over the five years between 2008 and 2013. Finally, this study highlights the need for population-based studies that track the prevalence of sight-impairing eye conditions and treatment effects over time.

## Additional file

Additional file 1: The economic impact of sight loss and blindness in the UK adult population – prevalence rates by age, gender, ethnicity and severity. This additional file provides a detailed overview of methods to estimate prevalence, any assumptions and the prevalence data sources underlying this research article. (DOCX 47 kb)

## Abbreviations

AMD: Age-related macular degeneration
BCVA: Best corrected visual acuity
DALY: Disability adjusted life year
HRG: Healthcare Resource Groups
NHS: National Health Service
ONS: Office for National Statistics
QALY: Quality adjusted life year
R&D: Research and development
UK: United Kingdom
VEGF: Vascular endothelial growth factor
VSL: Value of a statistical life
WHO: World Health Organization
YLD: Years of healthy life lost due to disability
YLL: Years of life lost due to premature mortality
