# Supplementary material for: The economic impact of sight loss and blindness in the UK adult population
Source: BMC Health Serv Res. 2018 Jan 30;18:63. doi: 10.1186/s12913-018-2836-0 (PMC5791217; doi:10.1186/s12913-018-2836-0)
Supplement: Additional file 1: — The economic impact of sight loss and blindness in the UK adult population – prevalence rates by age, gender, ethnicity and severity. This additional file provides a detailed overview of methods to estimate prevalence, any assumptions and the prevalence data sources underlying this research article. (DOCX 47 kb) [file 12913_2018_2836_MOESM1_ESM.docx]

# The economic impact of sight loss and blindness in the UK adult population – prevalence rates by age, gender, ethnicity and severity

A variety of data sources were utilised to estimate prevalence of sight loss and blindness by age, gender, ethnicity, region, severity and major cause. Importantly, there are no sources which identified prevalence for all of the specifications of interest. As such, it was necessary to combine sources using the methods described in this supplementary file. Due to the limitations of sources, the prevalence estimates have some uncertainty which is accounted for through sensitivity testing outlined in the full paper.

Ethnicity groupings were defined as per the population data, although ‘mixed’ and ‘other’ were combined into a single grouping ‘other’. Severity groupings were low vision (visual acuity (VA) of <6/12-6/18), partial sight (VA of <6/18-6/60) and blindness (VA of <6/60). Major causes were categorised into the six groups of age-related macular degeneration (AMD), cataract, diabetic retinopathy (DR), glaucoma, uncorrected refractive error (RE) and other.

## Prevalence of sight loss and blindness in the UK

### Sight loss and blindness in people aged 75 years and older

The total prevalence of sight loss and blindness for people aged 75 years and older was derived from Evans et al (2002), who estimated the prevalence of sight loss and blindness in people aged 75 years and older in Britain using the Medical Research Council trial of assessment and management of older people in the community. In this trial, data were obtained from 14,600 participants aged 75 years and older. Sight loss and blindness overall was defined as VA <6/18, low vision as VA <6/18 to 3/60, and blindness as VA <3/60. The prevalence of VA <6/12 was also presented for comparison with other studies.

Evans et al (2002) showed rates of sight loss and blindness increasing from 10.8% in those aged 75-79 years up to 53.1% in those aged 90 years and older. Their results have been reproduced in Table 1.

Table 1: Prevalence of sight loss and blindness for the UK population 75 years and older (binocular visual acuity <6/18)

| Age | Number | Prevalence | 95% CI |
| --- | --- | --- | --- |
| **Men** |  |  |  |
| 75-79 | 2,961 | 8.9 | 7.1 to 10.7 |
| 80-84 | 1,695 | 16.3 | 14.3 to 18.4 |
| 85-89 | 782 | 30.2 | 25.9 to 34.5 |
| 90-94 | 182 | 42.3 | 34.5 to 50.1 |
| **Women** |  |  |  |
| 75-79 | 3,937 | 12.3 | 10.4 to 14.2 |
| 80-84 | 2,907 | 22.1 | 19.0 to 25.2 |
| 85-89 | 1,537 | 37.9 | 33.9 to 41.8 |
| 90-94 | 599 | 56.4 | 51.0 to 61.9 |

Source: Evans et al (2002).

### 65-74 years – total sight loss and blindness

Van der Pols et al (2000) carried out VA measurements in the context of the national diet and nutrition survey (NDNS) of people aged 65 years or over. VA was measured in 1,362 NDNS participants who were not classified as mentally impaired. Sight loss was measured in 195 (14.3%) subjects with ‘low vision’ defined by the World Health Organization (WHO) criteria as VA <6/18 in the better eye and ‘sight loss’ defined according to US criteria is VA <6/12 and better than 6/60. For VA<6/18, prevalence was 1.8% in males 65-74 and 4.7% in females of this age – 2.5% for all 65-74 year old people. For VA<6/12, prevalence was 9.8% in the 65-74 age group.

These data were used to estimate the overall prevalence of sight loss and blindness and of low vision in the 65-69 and 70-74 age groups, adjusting downwards for the relative difference (1.37) between van der Pols et al (2000) and Evans et al (2002) in the 75+ groups – since van der Pols found higher prevalence in that group (60% in 85+ and 26% in 75-84 for VA<6/12 compared to the Evans et al (2002) finding of 26% in 85+ and 15% in 75-84).

### Under 65 – total sight loss and blindness

Total sight loss and blindness prevalence rates in the 40-65 age groups were derived from English and Scottish data from Charles (2007) and Charles (2011), together with relativities by age and gender from the Eye Disease Prevalence Research Group (EDPRG) international multi-site data (Congdon et al, 2004) applied to the older age groups as derived from UK sources. For example, Congdon et al (2004) showed a relativity of 0.88/1.47 or 60% between sight loss in the 60-64 group compared to the 65-69 group. With sight loss of 5.2% and 5.9% estimated in 65-69 year old males and females respectively in the UK, this ratio implies sight loss of 3.1% and 3.5% respectively in the 60-64 year old males and females.

Charles (2007) and Charles (2011) were used for the groups aged under 40 years (where there are no EDPRG data) and for severity splits (using weighted averages of the English and Scottish rates). Charles (2007) based their estimates on the two UK national prevalence studies by Evans et al (2002) and van der Pols et al (2000). These estimates were adjusted by relativities from the overall rates of sight loss and blindness (derived from the Evans et al (2002)-adjusted van der Pols (2000) data as described in the 65-74 age groups).

### Prevalence of sight loss and blindness by age and gender

Table 2 outlines the estimated prevalence of sight loss and blindness for the UK population and outlines the sources utilised to estimate the total prevalence rates.

Table 2: Prevalence of sight loss and blindness for the UK population by age and gender

| **Age** | **Male** | **Female** | **Persons** | **Sources** |
| --- | --- | --- | --- | --- |
| 0-4 | 0.1% | 0.1% | 0.1% | Charles (2007); Charles (2011) |
| 5-9 | 0.2% | 0.1% | 0.2% |  |
| 10-14 | 0.2% | 0.2% | 0.2% |  |
| 15-19 | 0.3% | 0.2% | 0.3% |  |
| 20-24 | 0.3% | 0.3% | 0.3% |  |
| 25-29 | 0.3% | 0.3% | 0.3% |  |
| 30-34 | 0.3% | 0.3% | 0.3% |  |
| 35-39 | 0.3% | 0.3% | 0.3% |  |
| 40-44 | 0.8% | 0.9% | 0.9% | Charles (2007); Charles (2011) combined with relative age gender distributions from Congdon et al (2004a) |
| 45-49 | 1.1% | 1.2% | 1.1% |  |
| 50-54 | 1.4% | 1.5% | 1.4% |  |
| 55-59 | 1.9% | 2.1% | 2.0% |  |
| 60-64 | 3.0% | 3.3% | 3.2% |  |
| 65-69 | 5.1% | 5.5% | 5.3% | van der Pols et al (2000) adjusted by relative difference between van der Pols and Evans in the 75+ age groups |
| 70-74 | 7.5% | 7.6% | 7.6% |  |
| 75-79 | 8.9% | 12.3% | 10.7% | Evans et al (2002) |
| 80-84 | 16.3% | 22.1% | 19.6% |  |
| 85-89 | 30.2% | 37.9% | 35.0% |  |
| 90+ | 42.3% | 56.4% | 52.4% |  |

## Prevalence by cause

Causes of sight loss and blindness by age and gender as published in Evans et al (2004a) were used, together with the overall prevalence of sight loss and blindness, to estimate prevalence of sight loss and blindness by age, gender and major cause in those aged 75 years and older. The raw rates from Evans et al (2004a) were adjusted downwards to account for comorbidities, because overall sight loss from the five main eye conditions and ‘other’ eye diseases cannot exceed 100% but still need to be ‘attributed’ (e.g.  90+ women in Table 3 for the major five causes are 20%+54%+24%+7%+1%>100%). ‘Other’ represented 7.4% after factoring down for comorbidities.

To estimate prevalence rates by cause for other age groups the following sources were used:

- Owen et al (2003), Owen et al (2012) and Friedman et al (2004) were used to estimate prevalence rates in younger age groups for AMD;
- Reidy (1998) and Congdon et al (2004a) were used to estimate the prevalence rates in younger age groups for cataract;
- Access Economics (2008a) and Kempen et al (2004) were used to estimate prevalence rates in younger age groups for diabetic diseases;
- Access Economics (2008b) and Friedman et al (2004) were used to estimate prevalence rates in younger age groups for glaucoma;
- other eye diseases were assumed to maintain a constant 7.4% of the overall UK sight loss and prevalence rates by age and gender group from Evans et al (2004a); and
- RE was estimated as the residual (leftover) of total sight loss and blindness after subtracting the other conditions.

Table 3 presents the prevalence of sight loss and blindness by age and cause.

Table 3: Prevalence of sight loss and blindness for the UK population by age and cause

| **Age** | **AMD** | **Cataract** | **DR** | **Glaucoma** | **RE** | **Other** | **Total** |
| --- | --- | --- | --- | --- | --- | --- | --- |
| 0-4 | - | - | - | - | 0.1% | 0.0% | 0.1% |
| 5-9 | - | - | - | - | 0.1% | 0.0% | 0.2% |
| 10-14 | - | - | - | - | 0.2% | 0.0% | 0.2% |
| 15-19 | - | - | - | - | 0.2% | 0.0% | 0.3% |
| 20-24 | - | - | 0.0% | - | 0.3% | 0.0% | 0.3% |
| 25-29 | - | - | 0.0% | - | 0.3% | 0.0% | 0.3% |
| 30-34 | - | - | 0.0% | - | 0.3% | 0.0% | 0.3% |
| 35-39 | - | - | 0.1% | - | 0.2% | 0.0% | 0.3% |
| 40-44 | - | 0.1% | 0.1% | 0.1% | 0.5% | 0.1% | 0.9% |
| 45-49 | - | 0.1% | 0.1% | 0.1% | 0.8% | 0.1% | 1.1% |
| 50-54 | - | 0.2% | 0.3% | 0.1% | 0.8% | 0.1% | 1.4% |
| 55-59 | 0.1% | 0.3% | 0.3% | 0.1% | 1.1% | 0.1% | 2.0% |
| 60-64 | 0.1% | 0.5% | 0.3% | 0.2% | 1.8% | 0.2% | 3.2% |
| 65-69 | 0.6% | 0.9% | 0.4% | 0.4% | 2.5% | 0.4% | 5.3% |
| 70-74 | 1.2% | 1.4% | 0.4% | 0.6% | 3.4% | 0.6% | 7.6% |
| 75-79 | 2.1% | 2.2% | 0.4% | 0.7% | 4.6% | 0.8% | 10.7% |
| 80-84 | 6.3% | 3.8% | 0.5% | 1.7% | 6.0% | 1.5% | 19.6% |
| 85-89 | 12.4% | 8.9% | 0.5% | 2.6% | 8.1% | 2.6% | 35.0% |
| 90 and over | 24.4% | 11.8% | 0.2% | 2.9% | 9.2% | 3.9% | 52.4% |

## Prevalence by severity

Splits between low vision, partial sight and blindness were based on Evans et al (2004a), using the relativities between the <6/18, 6/18-3/60 and <3/60 groups, together with a parameter estimating the proportion of blindness <6/60 relative to all sight loss and blindness (<6/12). This enabled a separation of those with VA<6/60 from those with VA<3/60 and a separation of VA<6/12-6/18. This parameter was based on two sources.

- Reidy et al (1998) separated sight loss and blindness severity into three groupings: <6/12-6/18, <6/18-6/60 and <6/60. Population prevalence of bilateral sight loss and blindness (<6/12) was around 30% and 92 of these 448 cases (21%) had VA <6/60 in one or both eyes. This 21% parameter was considered as the upper bound for the proportion of the 75+ population with VA<6/60 relative to those with with VA<6/12.
- Evans et al (2002) showed blindness measured as <3/60 as 2.1% and sight loss and blindness (<6/12) as 19.9% across the 75+ population. The ratio of these rates was necessarily a lower bound (10.6%).
- The average of the two estimates (15.8%) was used as the parameter for blindness as a share of total sight loss in the 75+ age group.

To derive severity splits for younger age groups it was necessary to use a number of sources:

- Owen et al (2003) and Owen et al (2012) were used to determine severity splits for AMD in younger age groups.
- Congdon et al (2004a), Owen et al (2003) and Access Economics (2004) were used to determine severity splits for cataract in younger age groups.
- Owen et al (2006), Coffey et al (1993), Friedman et al (2004) and Access Economics (2008b) were used to determine severity splits for glaucoma in younger age groups.
- Kempen et al (2004) and Access Economics (2008a) were used to determine severity splits for diabetic eye conditions in younger age groups.
- Kempen et al (2004a) and Access Economics (2004) were used to determine severity splits for refractive error.

Table 4 presents severity shares of total prevalence by age and cause, along with the sources and any adjustments or assumptions.

Table 4: Sight loss or blindness by severity, age and cause

| **Cause/ age** | **<6/12-6/18** | **<6/18-6/60** | **<6/60** | **Sources/methods** |
| --- | --- | --- | --- | --- |
| **AMD** | | | | |
| 55-59 | 100% | 0% | 0% | Owen et al (2003), Owen et al (2012) |
| 60-64 | 100% | 0% | 0% |  |
| 65-69 | 78% | 17% | 5% |  |
| 70-74 | 80% | 13% | 7% |  |
| 75-79 | 43% | 15% | 42% | <6/60 derived as 15.8% from Evans et al (2002) and Reidy et al (1998) multiplied by the share of all sight loss attributed to AMD from Evans et al (2004a), <6/18 - 6/60 and <6/12 derived as relative ratio from Evans et al (2004a) for all sight loss applied to estimated prevalence of <6/60 |
| 80-84 | 53% | 13% | 34% |  |
| 85-89 | 57% | 14% | 29% |  |
| 90+ | 63% | 13% | 25% |  |
| **Cataract** | | | | |
| 40-44 | 100% | 0% | 0% | Access Economics (2004) |
| 45-49 | 100% | 0% | 0% |  |
| 50-54 | 58% | 19% | 23% | Overall severity split from Reidy et al (1998) combined with over 75 estimates from Evans et al (2002) to solve for shares |
| 55-59 | 58% | 19% | 23% |  |
| 60-64 | 58% | 19% | 23% |  |
| 65-69 | 58% | 19% | 23% |  |
| 70-74 | 30% | 46% | 23% |  |
| 75-79 | 73% | 20% | 7% | <6/60 derived as 15.8% from Evans et al (2002) and Reidy et al (1998) multiplied by the share of all sight loss attributed to AMD from Evans et al (2004a), <6/18 - 6/60 and <6/12 derived as relative ratio from Evans et al (2004a) for all sight loss applied to estimated prevalence of <6/60 |
| 80-84 | 74% | 21% | 5% |  |
| 85-89 | 72% | 19% | 9% |  |
| 90+ | 77% | 18% | 5% |  |
| **DR** | | | | |
| 20-74 | 41% | 41% | 17% | Average of over 75+ shares |
| 75-79 | 28% | 28% | 44% | <6/60 derived as 15.8% from Evans et al (2002) and Reidy et al (1998) multiplied by the share of all sight loss attributed to AMD from Evans et al (2004a), <6/18 - 6/60 and <6/12 - 6/18 split equally |
| 80-84 | 38% | 38% | 23% |  |
| 85-89 | 49% | 49% | 1% |  |
| 90+ | 49% | 49% | 2% |  |
| **Glaucoma** | | | | |
| 40-44 | 55% | 17% | 28% | Average of over 60 shares |
| 45-49 | 55% | 17% | 28% |  |
| 50-54 | 56% | 17% | 28% |  |
| 55-59 | 56% | 17% | 28% |  |
| 60-64 | 4% | 35% | 60% | Access Economics (2008b) |
| 65-69 | 7% | 48% | 45% |  |
| 70-74 | 7% | 49% | 44% |  |
| 75-79 | 51% | 11% | 38% | <6/60 derived as 15.8% from Evans et al (2002) and Reidy et al (1998) multiplied by the share of all sight loss attributed to AMD from Evans et al (2004a), <6/18 - 6/60 and <6/12 derived as relative ratio from Evans et al (2004a) for all sight loss applied to estimated prevalence of <6/60 |
| 80-84 | 67% | 12% | 20% |  |
| 85-89 | 66% | 12% | 22% |  |
| 90+ | 65% | 9% | 27% |  |
| **RE** | | | | |
| All ages | 75% | 24% | 1% | Kempen et al (2004) and Access Economics (2004) |
| **Total sight loss and blindness** | | | | |
| 75+ | 64% | 20% | 16% | <6/60 derived as 15.8% from Evans et al (2002) and Reidy et al (1998), 6/60 or greater derived as sum of detailed causes outlined above |

## Prevalence by ethnicity group

Ethnicity splits were then applied to the overall prevalence rates – by age, gender and severity – based on relative risks for particular eye diseases (also by age, gender and severity), derived from literature.

Table 5 shows the relative risk of selected eye diseases due to ethnicity and the sources these relative risks were derived from. Total prevalence rates by ethnicity were calculated as the sum of prevalence from the relative risks multiplied against the initial prevalence estimates for the UK overall by condition and age/gender groups, and fitted back proportionally into the total population of people with sight loss and blindness. That is, the prevalence rates estimated by ethnicity were each respectively calibrated such that the sum of each ethnicity group after applying relative risks was equal to the total prevalence rates in the UK by age, gender and condition.

For other eye diseases, no robust differences in relative risk as a result of ethnicity have been found (Munier et al 1998; Ghafour et al 1983), so the relative risk was assumed to be one for each ethnicity group (i.e. the same as the overall rate). Moreover, where relative risks were not observed for other ethnic groups, the relative risk was assumed to be one for each age and gender group.

Table 5: Relative risk of selected eye diseases due to ethnicity

| Condition/age | Males | Females | Persons |
| --- | --- | --- | --- |
| **AMD** | Black:white (Friedman et al, 2004) | Black:white (Friedman et al, 2004) |  |
| 50-54 | 1.235 | 3.400 |  |
| 55-59 | 1.268 | 3.727 |  |
| 60-64 | 1.000 | 2.857 |  |
| 65-69 | 0.713 | 1.729 |  |
| 70-74 | 0.470 | 0.967 |  |
| 75-79 | 0.287 | 0.520 |  |
| 80+ | 0.131 | 0.149 |  |
|  | Asian:white (Das et al, 1994) | Asian:white (Das et al, 1994) |  |
| 70+ | 0.438 | 0.821 |  |
| **Cataract** | Black:white (Congdon et al, 2004) | Black:white (Congdon et al, 2004) | Asian:white (Das et al, 1994) |
| 40-49 | 0.607 | 1.158 | 11.000 |
| 50-54 | 0.918 | 1.460 | 8.167 |
| 55-59 | 0.927 | 1.362 | 8.167 |
| 60-64 | 0.862 | 1.189 | 2.300 |
| 65-69 | 0.781 | 1.029 | 2.300 |
| 70-74 | 0.711 | 0.912 | 1.453 |
| 75-79 | 0.663 | 0.843 | 1.453 |
| 80+ | 0.648 | 0.795 | 1.453 |
| **DR** | Black:white (Kempen et al, 2004) | Black:white (Kempen et al, 2004) |  |
| 40-49 | 1.450 | 1.917 |  |
| 50-64 | 1.222 | 2.124 |  |
| 65-74 | 0.621 | 1.417 |  |
| 75+ | 1.110 | 1.209 |  |
|  |  |  | Asian:white (Das et al, 1994) |
| All ages |  |  | 1.353 |
| **Glaucoma** | Black:white (Friedman et al, 2004) | Black:white (Friedman et al, 2004) |  |
| 40-49 | 1.528 | 1.819 |  |
| 50-54 | 2.803 | 2.517 |  |
| 55-59 | 3.600 | 2.804 |  |
| 60-64 | 4.186 | 2.967 |  |
| 65-69 | 4.415 | 2.937 |  |
| 70-74 | 4.238 | 2.727 |  |
| 75-79 | 3.710 | 2.388 |  |
| 80+ | 2.367 | 1.415 |  |
| **RE** | Black:white (Kempen et al, 2004a) | Black:white (Kempen et al, 2004a) |  |
| 40-49 | 0.614 | 0.430 |  |
| 50-54 | 0.604 | 0.493 |  |
| 55-59 | 0.551 | 0.539 |  |
| 60-64 | 0.468 | 0.572 |  |
| 65-69 | 0.373 | 0.591 |  |
| 70-74 | 0.281 | 0.600 |  |
| 75-79 | 0.202 | 0.604 |  |
| 80+ | 0.096 | 0.616 |  |

## Supplementary references

Access Economics. Clear Insight: The economic impact and cost of vision loss in Australia. Access Economics. Canberra. 2004.

Access Economics. The cost of partial sight and blindness in Canada. Report for Canadian National Institute for the Blind (CNIB) and Canadian Ophthalmology Society. Access Economics, Canberra. 2008.

Access Economics. The cost of partial sight and blindness in Japan. Report for Japan Ophthalmologists Association, National Institute of Sensory Organs, and Juntendo University WHO Collaborating Centre for the Prevention of Blindness. Access Economics. Canberra. 2008a.

Charles N. A brief guide to carrying out research about adult social care services for visually impaired people. National Institute for Health Research. 2011.

Charles N. Estimates of the number of older people with a partial sight and blindness in the UK. British Journal of Partial sight and blindness 2007;25(3):199-215

Congdon N, O'Colmain B, Klaver CCW, Klein R, Munoz B, Friedman DS, et al. Causes and Prevalence of visual impairment among adults in the United States. Archives of Ophthalmology 2004;122:477-485.

Congdon N, Vingerling JR, Klein BEK, West S, Friedman DS, Kempen J, et al. Prevalence of cataract and pseudophakia/aphakia among adults in the United States. Archives of Ophthalmology 2004a;122:487-494.

Das BN, Thompson JR, Patel R, Rosenthal AR. The prevalence of eye disease in Leicester: a comparison of adults of Asian and European descent. Journal of the Royal Society of Medicine 1994;87:219-222.

Desai P, Reidy A, Minassian, DC. National cataract surgery survey 1997–8: a report of the results of the clinical outcomes. British Journal of Ophthalmology 1999;83:1336–1340.

Evans JR, Fletcher AE, Wormald RPL, Siu-Woon Ng E, Sterling S, Smeeth L, et al. Prevalence of visual impairment in people aged 75 years and older in Britain: results from the MRC trial of assessment and management of older people in the community. British Journal of Ophthalmology 2002;86:795-800.

Evans JR, Fletcher AE, Wormald RPL. Causes of visual impairment in people aged 75 years and older in Britain: an add-on study to the MRC Trial of Assessment and Management of Older People in the Community. British Journal of Ophthalmology 2004a;88:365-370.

Friedman DS, O'Colmain BJ, Munoz B, Tomany MA, McCarty C, de Jong PTVM, et al. Prevalence of age-related macular degeneration in the United States. Archives of Ophthalmology 2004;122:564-572.

Friedman DS, Wolfs RCW, O'Colmain BJ, Klein BE, Taylor HR, West S, et al. Prevalence of open angle glaucoma among adults in the United States. Archives of Ophthalmology 2004a;122:532-538.

Ghafour IM, Allan D, Foulds WS. Common causes of blindness and visual handicap in the west of Scotland’, British Journal of Ophthalmology 1983;67:209-213.

Kempen JH, Mitchell P, Lee KE, Tielsch JM, Broman AT, Taylor HR, et al. The prevalence of refractive errors among adults in the United States, Western Europe and Australia. Archives of Ophthalmology 2004a;122:495-505.

Kempen JH, O'Colmain BJ, Leske C, Haffner SM, Klein R, Moss SE, et al. The prevalence of diabetic retinopathy among adults in the United States. Archives of Ophthalmology 2004;122:552-563.

Munier A, Gunning T, Kenny D, O’Keefe M. Causes of blindness in the adult population of the Republic of Ireland. British Journal of Ophthalmology 1998;82:630‑633.

Owen CG, Fletcher AE, Donoghue M, Rudnicka AR. How big is the burden of visual loss caused by age related macular degeneration in the United Kingdom? British Journal of Ophthalmology 2003;87:312-317

Owen CG, Jarra Z, Wormald R, Cook DG, Fletcher AE, Rudnicka AR. The estimated prevalence and incidence of late stage age related macular degeneration in the UK. British Journal of Ophthalmology 2012;96:752-756.

Reidy A, Minassian DC, Vafidis G, Joseph J, Farrow S, Wu J, et al. Prevalence of serious eye disease and visual impairment in a north London population: population based, cross sectional study. British Medical *Journal* 1998;316:1643-1646.

van der Pols JC, Bates CJ, McGraw PV, Thompson JR, Reacher M, Prentice A, et al. Visual acuity measurements in a national sample of British elderly people. British Journal of Ophthalmology 2000;84:165-170.
